# Assessing Cardiometabolic Health Risk in Children Living With Grandparent Primary Caregivers: Interim Analysis

**DOI:** 10.1093/geroni/igab046.3464

**Published:** 2021-12-17

**Authors:** MinKyoung Song, Laura Hayman, Karen Lyons, Hannah Bessette, Mary Roberts Davis, Kerri Winters-Stone, Carol Musil

**Affiliations:** 1 Oregon Health & Science University, Portland, Oregon, United States; 2 University of Massachusetts Boston, Boston, Massachusetts, United States; 3 Boston College, Chestnut Hill, Massachusetts, United States; 4 Case Western Reserve University, Cleveland, Ohio, United States

## Abstract

Minimal research has been conducted on the effect that grandparents as primary caregivers have on the cardiometabolic health of children who live with them, even though a number of studies have examined the influence of parent caregivers. As a first step towards filling that gap, we examined physiological and behavioral indicators of cardiometabolic health risk among children (aged 7 to 12 years) living with grandparent primary caregivers in Oregon and Washington. We measured body mass index and total cholesterol/glycohemoglobin (HbA1c), as well as physical activity/sleep and diet. In this preliminary analysis of our findings with 10 dyads (mean age 64.2 ± 4.0 years for grandparents; 9.3 ± 1.9 years for grandchildren), we report that on most of the indicators - obesity, physical activity, sleep, and diet - these children’s levels were comparable to national averages across all household types (not differentiated by type of family structure). However, 25% of the grandchildren (n=2) participating in our study had a total cholesterol level ≥ 200, compared to 7.4% of children from a nationally representative dataset. Similarly, 14% of the grandchildren (n=1) participating in our study had HbA1c ≥ 6.5%, compared to < 0.5% of children from a nationally representative dataset. Our findings suggest that these children may be at higher cardiometabolic health risk (e.g., hyperlipidemia). Further investigations with a larger sample and more examination of cardiometabolic risk profiles including lipids/blood glucose assessment are required to validate our preliminary findings.

